# The Potential Clinical Applications of a Microfluidic Lab-on-a-Chip for the Identification and Antibiotic Susceptibility Testing of *Enterococcus faecalis*-Associated Endodontic Infections: A Systematic Review

**DOI:** 10.3390/dj12010005

**Published:** 2023-12-26

**Authors:** Carlos M. Ardila, Gustavo A. Jiménez-Arbeláez, Annie Marcela Vivares-Builes

**Affiliations:** 1 Basic Studies Department, School of Dentistry, Universidad de Antioquia UdeA, Medellín 050010, Colombia; 2 School of Dentistry, University Institution Visión de Las Américas, Medellín 050031, Colombia; gustavo.jimenez@uam.edu.co (G.A.J.-A.); anny.vivares@uam.edu.co (A.M.V.-B.)

**Keywords:** *Enterococcus faecalis*, dental pulp diseases, periapical diseases, microfluidic lab-on-a-chips, clinical efficacy

## Abstract

This systematic review evaluated the potential clinical use of microfluidic lab-on-a-chip (LOC) technology in the identification and antibiotic susceptibility testing of *E. faecalis* in endodontic infections. The search methodology employed in this review adhered to the PRISMA guidelines. Multiple scientific databases, including PubMed/MEDLINE, SCOPUS, and SCIELO, were utilized, along with exploration of grey literature sources. Up to September 2023, these resources were searched using specific keywords and MeSH terms. An initial comprehensive search yielded 202 articles. Ultimately, this systematic review incorporated 12 studies. Out of these, seven aimed to identify *E. faecalis*, while the remaining five evaluated its susceptibility to different antibiotics. All studies observed that the newly developed microfluidic chip significantly reduces detection time compared to traditional methods. This enhanced speed is accompanied by a high degree of accuracy, efficiency, and sensitivity. Most research findings indicated that the entire process took anywhere from less than an hour to five hours. It is important to note that this approach bypasses the need for minimum inhibitory concentration measurements, as it does not rely on traditional methodologies. Microfluidic devices enable the rapid identification and accurate antimicrobial susceptibility testing of *E. faecalis*, which are crucial for timely diagnosis and treatment in endodontic infections.

## 1. Introduction

*Enterococcus faecalis*, a Gram-positive coccus that can thrive in both oxygen-rich and oxygen-deprived conditions, is a commonly encountered microorganism within the digestive tracts of healthy individuals. This microorganism can be found in a variety of environments, including soil, water, and food products [1]. *E. faecalis* stands out as the most predominant among enterococcal species, as it is responsible for approximately 80% to 90% of hospital-acquired infections attributed to enterococci. Operating as an opportunistic pathogen, *E. faecalis* seizes the opportunity to cause severe diseases, occasionally leading to fatal outcomes, especially when the host’s immune defences are compromised [2]. Moreover, it is involved in the development of chronic infections like infective endocarditis, abdominal infections, urinary tract infections, surgical wound infections, bacteraemia, and endodontic infections [3].

Microbial analysis of endodontic infections demonstrated that the composition of the microbiota was not consistently specific but varied in abundance based on the clinical diagnosis [4]. Primary endodontic infections occur when microorganisms invade and establish colonies in necrotic root canals, whereas secondary and persistent infections arise due to microorganisms entering the root canals either because of professional dental procedures or by surviving chemo-mechanical cleaning procedures and persisting within the root canal environment [5,6]. *E. faecalis* is often found in the root canals of teeth with persistent or recurrent endodontic infections. This microorganism is particularly problematic in endodontics because it could survive and thrive in the harsh environment of the root canal system, even in the presence of antimicrobial treatments. *E. faecalis* is known for its ability to form biofilms within the root canal system, which can protect it from antimicrobial agents and the host’s immune response [5,7]. This biofilm formation contributes to its persistence in endodontic infections. Its resistance to disinfection and ability to persist in the root canal can make it challenging to eliminate infected teeth [5,8]. *E. faecalis* has been recognized as the predominant species found in the root canals of teeth with endodontic failure through both biochemical assessments and molecular analysis [8,9].

Traditionally, the detection of microorganisms in endodontic samples relied on conventional culture techniques, which encompassed the isolation, cultivation, and identification of microbes based on their morphological characteristics and biochemical tests. The utilization of commercial kits for bacterial identification helps circumvent several challenges associated with interlaboratory discrepancies, including inconsistencies in conditions and reagents [10]. Nonetheless, the culture method might underestimate the occurrence of certain oral pathogens, as it could falter in isolating and cultivating certain bacteria, particularly those with the most demanding growth requirements. Furthermore, any characteristic associated with the phenotype can result in challenges during identification, potentially even resulting in misidentification, as incomplete data may lead to one species being erroneously attributed to another [11]. To address the constraints posed by culture and biochemical identification methods, molecular techniques such as gene sequencing and polymerase chain reaction (PCR) have provided an enhanced comprehension of the profile of endodontic infections [12]. While molecular techniques have several advantages for identifying *E. faecalis* in endodontic infections, they also come with some drawbacks. For instance, these methods demand a higher level of technical expertise and specialized training, both in terms of sample preparation and data analysis [13,14]. Additionally, they can be sensitive to contamination, which may result in false-positive results. Conversely, false negatives can occur when the target DNA is either insufficient in quantity or degraded in the sample. Molecular techniques, such as PCR and gene sequencing, can also be more expensive than traditional culture and biochemical methods [14]. The costs associated with acquiring specialized equipment and reagents can be prohibitive for certain laboratories or clinics. Moreover, there can be variations in the selection of primers, protocols, and techniques among different laboratories, leading to inconsistencies in results and hindering comparisons between studies [13,14]. Thus, while molecular techniques are generally faster than traditional culture methods, they still require more time than some rapid diagnostic tests. Similarly, traditional antibiotic susceptibility testing (AST) methods yield reliable results but are time-consuming and labour-intensive [13]. Consequently, therapeutic intervention with empirical antimicrobial therapy comes before understanding of AST, and clinicians tend to administer antimicrobials with the broadest spectrum of activity and the greatest feasible dosage [14,15]. Therefore, there is a definite need for novel techniques that enable rapid, low-cost, and simply adopted AST without sacrificing accuracy [15].

The recent advancements in lab-on-a-chip (LOC) technology have the potential to address these challenges, improve fundamental bacteriological investigation, and contribute to the advancement of therapeutic methodologies. LOC technology enables the miniaturization and automation of complex laboratory processes, allowing for the rapid and efficient analysis of biological, chemical, or environmental samples [15,16]. Microfluidic systems are a subset of LOC devices, but an LOC system may also include other components beyond microfluidics, such as sensors, detectors, or actuators, to create a complete analytical or diagnostic platform [17]. Microfluidics specifically focuses on the manipulation and control of small volumes of fluids (usually in the microlitre to nanolitre range) within microchannels or microstructures. Microfluidic systems have also proven to be highly valuable tools for the identification of microorganisms due to their versatility, precision, and efficiency [18]. LOC and microfluidic systems can provide rapid results, often within minutes, whereas biochemical assessments and molecular analysis can take hours or even days [16,17]. Moreover, LOC and microfluidic systems can detect low concentrations of bacteria, making them more sensitive than some biochemical tests [15]. This is crucial for identifying *E. faecalis*, which may be present in low numbers in some clinical samples.

Considering all the aforementioned factors, the aim of this systematic review is to assess the potential clinical application of microfluidic lab-on-a-chip technology in the identification and antibiotic susceptibility testing of *E. faecalis* in endodontic infections.

## 2. Materials and Methods

### 2.1. Search Approach

The search methodology employed in this systematic review adhered to the PRISMA (Preferred Reporting Items for Systematic Reviews and Meta-analyses) guidelines [19]. This investigative approach involved the utilization of multiple scientific databases, namely PubMed/MEDLINE, SCOPUS, and SCIELO, alongside the exploration of grey literature sources. Up until September 2023, the exploration of these resources involved the deployment of keywords and MeSH terms encompassing “microfluidics”, “organ on a chip”, “lab-on-a-chip”, “micro physiological systems”, “microchip platforms”, “sensors”, “bioassays”, “antimicrobial susceptibility test”, “biofilms”, and “*E. faecalis*”, and encompassed studies published in all languages. Subsequently, we conducted database searches employing Boolean operators (AND, OR) to combine the terms “microfluidics”, “organ on a chip”, “lab-on-a-chip”, “micro physiological systems”, “microchip platforms”, “sensors”, “bioassays”, “antimicrobial susceptibility test”, “biofilms”, and “*E. faecalis*”. The systematic review protocol was formally registered on the Open Science Forum Database under the following identifier: Protocol: osf.io/wv9c4.

### 2.2. Selection Criteria

The inclusion criteria for this assessment encompassed studies employing microfluidic platforms or LOC technologies, in conjunction with the utilization of 3D printing and/or bioprinting techniques for the development of organ-on-a-chip systems. The selected studies should aim to identify and/or test the antibiotic susceptibility of *E. faecalis.* In contrast, systematic reviews, meta-analyses, abstracts, narrative reviews, case reports, brief communications, conferences, patents, and studies deficient in essential details regarding the fabrication process were excluded from consideration.

### 2.3. Question

The primary objective of this systematic review is to answer the question: Can microchip platforms enable the identification and antibiotic susceptibility testing of *E. faecalis*?

P: investigations involving *E. faecalis*.

I: utilization of microfluidic LOC technology.

C: comparative control experiments.

O: identification and antibiotic susceptibility testing of *E. faecalis*.

### 2.4. Review Course

Two investigators assessed the titles and abstracts of articles for potential inclusion. In instances where discrepancies in study selection arose, a third author (GJ) was available to facilitate resolution. The level of agreement among observers, with a threshold set at >91, was assessed through the application of the Kappa statistical test to establish significance.

### 2.5. Compilation of Data

The relevant data extracted from the selected investigations were organized within a tabular format. This procedure was conducted autonomously by each investigator. Subsequently, a comparative analysis was undertaken to align the information. The data encompassed details pertaining to the utilization of the microfluidic LOC device, along with crucial attributes like the composition of materials employed in its fabrication, culture methodologies, microbial strains, growth conditions, and principal research findings.

### 2.6. Risk of Bias

The assessment of methodological quality in the incorporated studies was conducted employing the quality appraisal tool devised for in vitro research [20]. A comprehensive evaluation encompassed the study’s purposes, sampling approach, sample size, features of the comparison group, thorough explanation of the investigate methodology, operator stipulations, randomization procedures, measurement of outcomes, factors affecting results, blinding processes, management of outcomes, and statistical analyses (12 factors). Each report was ascribed a general quality score derived from the cumulative score range, categorized as either low (>70%), medium (50–70%), or high (<50%).

## 3. Results

**Figure 1:**
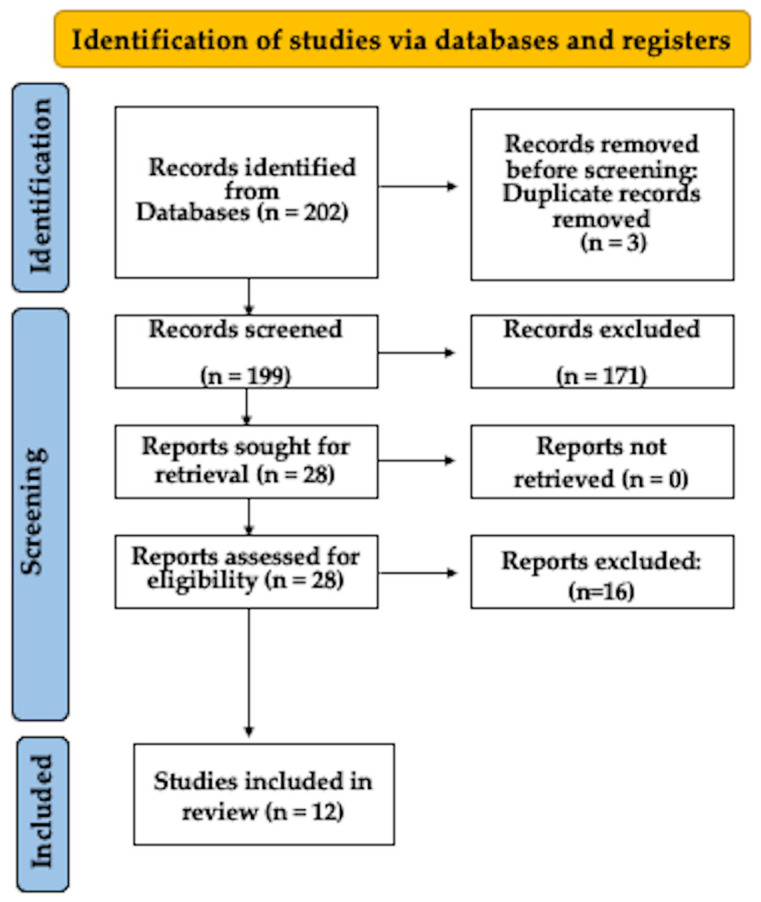
Flowchart depicting the study selection method.

A comprehensive initial search yielded a total of 202 articles. Subsequently, 171 publications were eliminated from consideration due to their lack of focus on *E. faecalis* or their investigation of genes or proteins. Furthermore, three duplicate papers were excluded. Following a thorough examination of the complete texts, an additional 16 studies were excluded as they did not align with the predefined selection criteria. Ultimately, this systematic review incorporated 12 studies [21,22,23,24,25,26,27,28,29,30,31,32], as illustrated in Figure 1.

The characteristics of the studies encompassed in the analysis are detailed in Table 1. The publications incorporated in this review span the timeframe from 2009 [21] to 2022 [31,32].

Table 1 shows that out of the 12 studies assessed in this systematic review, 7 aimed to identify *E. faecalis* [21,24,25,27,29,31,32], while the remaining 5 evaluated its susceptibility to different antibiotics [22,23,26,28,30]. In all the studies, it was observed that the newly developed microfluidic chip significantly reduces the detection time when compared to traditional methods. This enhanced speed comes with a high degree of accuracy, efficiency, and sensitivity. Most research findings indicated that the entire process took anywhere from less than an hour to five hours, and the outcomes were just as precise as those obtained using the guidelines set by the Clinical & Laboratory Standards Institute (CLSI) [33] and the European Committee on Antimicrobial Susceptibility Testing (EUCAST) [34]. The utilization of this microfluidic approach significantly reduced the standard diagnostic time, which typically exceeds 24 h in conventional, culture-based detection methods. This method could potentially eliminate the need for pre-incubation since tests can be performed on an individual microorganism. Additionally, it was noted that this approach circumvents the necessity for minimum inhibitory concentration (MIC) measurements, as it does not rely on traditional methodologies. Thus, these findings offer valuable insights for clinical screening purposes.

Table 1 further shows that a range of resources and microfabrication procedures can be used to create a microfluidic LOC for studying *E. faecalis.* Photolithography, for example, can be used to create silicon wafers with nanoscale chip features. These microfluidic chips often include compartments such as microchannels, chambers, and reservoirs as well as functional components for precise liquid manipulation such as pumps, mixers, and valves. According to the review results, polydimethylsiloxane (PDMS) silicone rubber is a regularly utilized material in laboratory settings for the construction of such devices.

In this systematic review, various strains of *E. faecalis* were investigated, and many of the tests were conducted in accordance with the recommendations of CLSI [33] and EUCAST [34], with minimal deviations.

*E. faecalis* susceptibility to ampicillin, oxacillin, gentamicin, vancomycin, tetracycline, and kanamycin was assessed. Most studies also took into consideration the MIC ranges defined by CLSI [33] and EUCAST [34], and the microfluidic LOC was employed for the simultaneous evaluation of multiple antibiotics. It is worth noting that microfluidic systems operate with microlitre volumes, in contrast to traditional techniques that use millilitre volumes.

Analysing the dynamics of microfluidic lab-on-a-chip systems for the identification and antibiotic susceptibility testing in *E. faecalis* involves a nuanced examination of speed, precision, and complexity.

Microfluidic systems can offer rapid results due to their small-scale and efficient fluid handling. Quick identification and antibiotic susceptibility testing are critical for timely intervention and treatment decisions. However, high-speed processes might sacrifice the thoroughness of analysis, and some rapid techniques may provide quick results but may compromise on the depth of information obtained.

Microfluidic devices can achieve high precision by precisely controlling sample volumes and reaction conditions. Accurate identification and susceptibility results are crucial for effective treatment. Nevertheless, enhanced precision may involve more complex and time-consuming procedures. Striking a balance between speed and precision is essential to avoid sacrificing one for the other.

Finally, microfluidic lab-on-a-chip systems offer miniaturization and automation, reducing the overall complexity compared to traditional methods. The integration of multiple functions into a single device streamlines the testing process.

Nonetheless, increasing complexity may lead to challenges in device fabrication, operation, and maintenance. Simplicity is often sacrificed for added features, potentially impacting the user-friendliness of the system.

In summary, achieving an effective microfluidic lab-on-a-chip system for *E. faecalis* testing involves carefully navigating the trade-offs between speed, precision, and complexity. The optimal design will depend on the specific requirements of the testing scenario and the priorities of the healthcare application.

All the investigations were deficient in terms of essential aspects, such as the calculation of sample size, clinician standardization, blinding, and randomization procedures. Nonetheless, they demonstrated adequate detail in defining aims, incorporating a control group, describing the experimental technique, presenting data analysis, and reporting outcomes. Therefore, 10 studies had a score of 58%, and the remaining 2 had a score of 67% [31,32], signifying a medium risk of bias according to the rating tool used [20] (Table 2). Additionally, the purposes, conceptual outlines, methods, and result variables differed among the individual models, rendering aggregated quantitative analyses challenging.

## 4. Discussion

Although traditional culture, PCR, and molecular approaches can be used to detect *E. faecalis*, their routine diagnostic value is restricted due to intrinsic drawbacks. Furthermore, the emergence of bacterial antibiotic resistance highlights the importance of developing rapid, high-throughput, and accurate methods to assess microbial susceptibility to various antibiotics that are easily accessible to clinical microbiology laboratories. As observed in this systematic review, the utilization of microfluidic chips enables the simultaneous, visual, and rapid detection of *E. faecalis* in a user-friendly manner, potentially enhancing the device’s efficiency and applicability for on-site rapid screening applications.

In this context, a transparent 3D microfluidic model replicating the root canal architecture of a central incisor was recently constructed using PDMS micro-moulding of a silicon master prepared with standard photolithographic techniques. Its optical transparency renders it exceptional for the real-time observation of fluorescently labelled particle trajectories and deposition kinetics [35]. Additionally, a microfluidic system was recently developed to mimic the biomaterial–dentin–pulp interface. The study demonstrated that it was possible to mimic various treatments on both healthy and diseased dentin while examining the structural arrangement of the bacterial community, cell shapes, and biomaterials. [36,37]. Also, an investigation utilized a microfluidic system to cultivate *E. faecalis* biofilms under continuous shear flow, assessing the bactericidal impact of highly acidic electrolyzed water on this microorganism. The microfluidic system, by offering conditions akin to those in naturally occurring biofilms, including a constant nutrient supply and shear flow, can potentially offer more accurate insights into the clinical bactericidal effect [38].

By manipulating fluid movement in the microfluidic chip, microfluidic technology unifies sample planning, response, separation, and identification. Microfluidic systems provide various advantages over traditional macro-scale procedures. The small dimension of the microchannel allows liquid flow to be directed by a constant laminar flow, needs fewer samples, and effectively controls temperature and mass transmission [39]. Founded on these benefits, the combination and incorporation of loop-mediated isothermal amplification (LAMP) identification of different microorganisms and microfluidic chips may offer a promising application opportunity for nucleic acid recognition and exploration. As reported in this systematic review, the microfluidic chip approach enabled the rapid identification of *E. faecalis* [32]. Similarly, a portable device for bacteria identification was designed, integrating droplet generation technology and LAMP on a microfluidic centrifugal disc. This device effectively detected *E. faecalis* in water samples within just 1 h, with a straightforward one-button activation, demanding less than 5 min of hands-on operation [31]. This method has significantly enhanced the velocity, sensitivity, and precision of bacterial identification in both clinical and environmental models when compared to traditional culture-based approaches [40].

Recent research indicates that microfluidic tools are capable of accurately quantifying microorganisms using digital techniques [41], streamlining standard AST assays that rely on dilution methods, and estimating the MIC based on microbial growth within droplets [42]. These systems provide precise liquid manipulation and rapid bacteria detection, potentially leading to improved MIC measurement exactitude. Therefore, droplet digital analyses offer a possibility that allows for the concurrent digital measurement of the initial culture density and the performance of susceptibility tests [30]. As outlined in this systematic review, microfluidic AST yielded results for *E. faecalis* within a range of 30 min [28] to 5 h [30]. Likewise, various studies demonstrated results for Escherichia coli within the time frame of 15 min [43] to 3.5 h [44], which depended on the microbial doubling time and the specific analytical method used.

Incorporating multiple experimental procedures into a unified platform can also diminish the need for researchers to make numerous adjustments, thereby reducing the risk of contamination and methodological variability [45]. As demonstrated in this study, droplet-based devices serve as a prime illustration of the integrative capabilities of microfluidics [46]. These timesaving and minimized technical handling aspects are unquestionably crucial factors to consider when using microfluidic devices in a clinical environment [45].

As demonstrated in this systematic review, it is essential to highlight that while conventional methods are constrained to analysing samples on flat 2D surfaces like culture flasks, Petri dishes, or well plates, LOC technology introduces a novel array of materials, with one of the most notable being polydimethylsiloxane [45]. This material is well suited for cellular and microbiological cultures due to its biocompatibility and gas permeability. Furthermore, its optical transparency facilitates microscopic observation and analysis. An important feature to note is its versatility, as it can be combined with various other materials, resulting in a wide range of device compositions. Consequently, microfluidics enables the operation of distinct stimuli, including fluid flow or gradient generation, in diverse or uniform three-dimensional scenarios [45,47].

The difference in volume and *E. faecalis* populations used in microfluidics versus conventional techniques, as detailed in this systematic review, is also an essential issue to consider [45]. Microfluidics operates with microlitre volumes, while traditional techniques work with millilitre measurements. This tiny volume converts microfluidic techniques into portable devices, enabling reagent, resource, and area cost savings, including detailed control of the analysis’s biological and physical characteristics [16,45]. The number of microorganisms in a LOC is decreased to hundreds or even a single microorganism, yielding more precise outcomes on a single-cell measure [16,45].

The utilization of LOC microfluidics in the identification and antimicrobial susceptibility testing of *E. faecalis* holds significant clinical relevance and implications for both patient care and healthcare systems. LOC technology enables the swift identification of *E. faecalis*, providing clinicians with timely diagnostic information. Rapid diagnostics are crucial for initiating prompt and targeted treatment strategies, reducing the time between identification and intervention. Microfluidic platforms offer precise antimicrobial susceptibility testing, allowing for tailored and effective antibiotic prescriptions. Enhanced precision aids in avoiding the misuse of antibiotics, minimizing the risk of antibiotic resistance, and optimizing patient outcomes. Moreover, point-of-care testing reduces turnaround times, streamlines decision making, and enhances overall patient management in various healthcare settings. The miniaturized nature of microfluidic systems requires smaller sample volumes and reagents, contributing to resource efficiency.

Therefore, reduced resource consumption can have economic implications for healthcare systems, making diagnostics more accessible and cost-effective.

Microfluidics LOC platforms offer numerous advantages in the identification and antimicrobial susceptibility testing of *E. faecalis*. However, they also come with certain limitations. Developing and operating microfluidic devices can be technically challenging, requiring specialized knowledge and equipment. This complexity can limit their accessibility and widespread use, particularly in resource-limited settings [15,16]. The lack of standardized protocols and assays for microfluidic devices can make it challenging to compare results across different platforms and studies [16]. Skilled personnel are required for operating and maintaining microfluidic devices, which can be a limiting factor in some clinical settings [15,16]. Despite these limitations, ongoing research and technological advancements continue to address many of these challenges, making microfluidics LOC platforms increasingly valuable tools in the field of microbiology and antimicrobial susceptibility testing.

This systematic review has several limitations. The existing data were derived from a limited number of in vitro studies, which may have limited therapeutic relevance. Additionally, these studies varied in their objectives, methodologies, and designs, leading to significant heterogeneity. The included studies also carried a moderate level of risk, but they do provide the potential to identify and assess antibiotic susceptibility in *E. faecalis.* Moderate-risk studies might have limitations that affect the generalizability of the results. This can impact the applicability of the findings to broader populations or settings. However, identifying moderate-risk areas in the existing literature can guide recommendations for future research. This may involve emphasizing the need for high-quality studies to address gaps or uncertainties.

In summary, LOC microfluidics is like a tiny, efficient lab that helps identify and assess how susceptible bacteria like *E. faecalis* are to antibiotics. Instead of relying on larger, more complex methods, these small devices provide quick and accurate results. They benefit patients by giving doctors faster and more informed treatment decisions. Additionally, these advances can make testing more accessible and affordable, improving healthcare for a wider range of people.

## 5. Conclusions

Microfluidics LOC platforms hold significant promise for various applications in the identification and antimicrobial susceptibility testing of *E. faecalis* in endodontic infections. Microfluidic devices can facilitate the rapid identification of *E. faecalis*, which is essential for timely diagnosis and treatment in endodontic infections. These platforms can be used to perform quick and accurate antimicrobial susceptibility tests, aiding in the selection of appropriate antibiotics for treatment. Microfluidic devices can be designed for use at the point of care, enabling dentists and endodontists to perform on-site testing, resulting in faster treatment decisions. Microfluidic systems can minimize sample volumes required for testing, which is especially valuable in endodontic procedures where limited sample availability can be a challenge. While microfluidics LOC platforms offer great potential for improving the diagnosis and treatment of *E. faecalis* in endodontic infections, their successful implementation requires rigorous research, development, and validation to ensure their effectiveness and reliability in clinical practice.

## Figures and Tables

**Table 1:** Main findings.

Materials Used	Culture, Bacterial Strains, and Growing Conditions	Main Results	Reference
The microchannels were generated through the process of hot embossing, utilizing a nickel–cobalt electroplated mould that was fabricated based on a silicon master template.	The PCR amplification of the extracted DNA was evaluated through the introduction of *E. faecalis* (ATCC 29212) into. *E. faecalis* was cultivated in 3 mL of brain heart infusion media at a temperature of 37 degrees Celsius for a duration of 16 to 18 h, with agitation at 250 revolutions per minute.	The genomic DNA of *E. faecalis* was successfully isolated and identified in microlitre-scale human whole-blood samples. The entire extraction process required less than an hour and can be further reduced by modifying the channel design and the pump setup.	[21]
The devices were manufactured employing soft lithography techniques. Specifically, masters were created by applying SU-8 photoresist (Microchem) onto silicon wafers through the process of photolithography.	Off-chip minimum inhibitory concentration assays were conducted in accordance with the Clinical and Laboratory Standards Institute protocols. Cell suspensions of *E. faecalis* (strain 1131) were prepared in 13 × 100 mm tubes by adjusting the cell concentration from overnight cultures to match the turbidity of a 0.5 McFarland standard, approximately ~108 CFU/mL, using cation-adjusted Mueller–Hinton broth.	Through the utilization of a microfluidic device, the MIC of vancomycin, tetracycline, and kanamycin against *E. faecalis* was determined, and the obtained MIC values were found to be consistent with those derived from conventional liquid broth dilution techniques. Notably, it was observed that bacterial cultures within the microfluidic devices achieved their MIC values more rapidly when compared to off-chip assays.	[22]
The microfluidic devices were made via hot roll lamination, which allows for quick, parallel, and cost-effective production. A colorimetric colour change is the simplest approach to confirm bacterial growth in a microfluidic chip.	The gel was produced with 20 mg/mL lysogenic broth for bacterial testing. On lysogenic broth agar plates, *E. faecalis* (DSM 16440) was grown. A colony was diluted in 10 mM phosphate-buffered saline to make the samples.	Ampicillin >1 µg/mL inhibited *E. faecalis* growth and Gentamicin resistance was observed at all doses. The quick microfluidic method reliably assesses susceptibility with MIC concentrations in agreement with standard reference methods.	[23]
This platform employs the centrifugal force generated by rotation to effectively trap bacteria directly from a liquid suspension within a chip. This is a polymeric microfluidic system.	*E. faecalis* ATCC 29212 was grown on agar at 37 °C for one night.	The entire process, from sample preparation to obtaining valuable results, takes approximately 1 h. This represents a substantial reduction in diagnostic time compared to the typically lengthier period of 24 h or more required for standard microbiological methods.	[24]
A layer of silica nanoparticles was applied to a surface made of polymethylmethacrylate and other thermoplastics.	*E. faecalis* (ATCC 47077) was introduced into 10 mL of sterile brain heart infusion medium that had been purged with nitrogen. The culture was allowed to incubate overnight, reaching the stationary phase, and subsequently stored at 4 °C for use within 24 h. On the microchip, droplets containing nickel cations, combined with bacteria and resazurin, were created.	The detection of metabolic inhibition in *E. faecalis* occurred within a 5 min timeframe. The entire procedure, which included the sequential injection of reagents and the simultaneous monitoring of droplet fluorescence intensity, was conducted directly on the microchip.	[25]
The silicon wafer was imprinted with mould using the established soft lithography method.	*E. faecalis* 24 was agitated and cultured in a brain heart infusion (BHI) medium for 12 h at 37 °C within a 2% BHI broth environment. An imaging platform was employed to measure the fluorescence intensity generated in the bacterial culture medium because of the redox reaction involving resazurin.	The microfluidic platform exhibited quicker performance, completing its task in 1 to 3 h, as opposed to the conventional gold standard, broth microdilution, which typically takes 12 to 18 h. Despite the speed, it maintained a similar level of accuracy. Ampicillin, kanamycin, and gentamicin were all effective against *E. faecalis.*	[26]
Masters were created in SU-8 photoresist (Microchem) on silicon wafers through the process of photolithography.	For *E. faecalis* identification, the bacteria were cultured at 37 °C overnight on Luria–Bertani (LB) agar. Afterward, isolated and selected individual colonies were transferred to a 100 mL solution of 25% LB. It homogenized the inoculum through vortexing and subsequently introduced 20 mL of cell suspension into a BacChip.	This automated microfluidic system can identify *E. faecalis* within a timeframe of less than 4 h.	[27]
The production of the microfluidic device primarily comprises the photolithography process for creating the SU-8 master template, soft lithography to produce polydimethylsiloxane replicas, and subsequently, bonding the device to microscope slides through plasma treatment.	*E. faecalis* (ATCC 29212) was cultivated in brain heart infusion agar/broth, following the procedures specified by ATCC. The growth of the bacteria was observed in a minimum of 100 droplets, utilizing time-lapse imaging, over a period of 2 h, with images captured at 15 min intervals.	The MIC derived through phenotypic analysis within droplets correlated with the MIC results obtained via the conventional broth microdilution method. Nonetheless, this method is notably swifter (30 min versus 16 to 24 h). All oxacillin concentrations greatly suppressed *E. faecalis* growth.	[28]
A microfluidic-based high-throughput qPCR assay was created. The entire primer design process, from fetching bacterial genome data to vetting primer candidates for quality, was automated.	Strains preserved in the Agroscope Culture Collection at −80 °C within sterile skim milk powder were reawakened and grown as per previously defined conditions.	The SpeciesPrimer pipeline was finalized within a timeframe of two to eight hours. SpeciesPrimer streamlines the process of primer design for precise species quantification, enabling a swift and precise quantitative analysis of *E. faecalis.*	[29]
Polydimethylsiloxane was poured over a polycarbonate master created through CNC milling and then incubated at 70 °C for a duration of 2 h.	*E. faecalis* (ATCC 51299) was cultured on agar plates containing 2% MH broth and then incubated at 37 °C overnight.	*E. faecalis* was susceptible to ampicillin. It was possible to significantly cut down the assay time to approximately 5 h, a notable improvement compared to the 20 h required by the conventional culture-based test.	[30]
The microfluidic centrifugal disc comprises four fundamental layers constructed from polycarbonate.	*E. faecalis* (ATCC 29212) was grown overnight in Luria–Bertani broth at 37 °C. Following that, the culture was serially diluted in deionized water and used for loop-mediated isothermal amplification, either in tubes or on a centrifuge disc.	The prototype device can detect *E. faecalis* in water samples by just pressing a start button, and the process takes 1 h with a total hands-on time of less than 5 min.	[31]
The LAMP-microfluidic chip was created using a mix of loop-mediated isothermal amplification (LAMP) and microfluidic technology.	Lyophilized standard strains (*E. faecalis* CGMCC 1.10682) and cryopreserved clinical isolates were cultured on appropriate agar plates before being picked for rejuvenation. Three repeated detections on three random clinical samples with positive results identified by LAMP-microfluidic chip were performed.	The disclosed LAMP-microfluidic chip approach can identify *E. faecalis* quickly, and the entire procedure from DNA extraction to amplification completion took just about 90 min, with detection sensitivities of less than 105 CFU/mL or copies/mL.	[32]

MIC = minimal inhibitory concentration.

**Table 2:** Risk of bias of the assessed investigations.

* Criteria Met	^ Points	^+^ Score	Reference
8	14	58%	[21]
8	14	58%	[22]
8	14	58%	[23]
8	14	58%	[24]
8	14	58%	[25]
8	14	58%	[26]
8	14	58%	[27]
8	14	58%	[28]
8	14	58%	[29]
8	14	58%	[30]
9	16	67%	[31]
9	16	67%	[32]

* Clear objectives, determination of sample size, sample technique, comparison group, detailed methodology, operator details, randomization, results measurement, outcome assessor details, blinding, statistical analysis, and presentation of results. ^ Points: adequately specified = 2 points, inadequately specified = 1 point, not specified = 0 point, and not applicable = exclude criteria from calculation. ^+^ Score = total points × 100/2 × number of criteria applicable.

## Data Availability

The data obtained in this review were pooled from the investigations included.
